# Multiparametric MRI-based radiomics model integrating tumor lesion and periprostatic adipose tissue for predicting bone metastasis in prostate cancer

**DOI:** 10.3389/fonc.2026.1918323

**Published:** 2026-09-07

**Authors:** Qian Zheng, Ruihong Chen, Yuying Xie, Sha Cui, Wenjin Bian, Jianting Li, Limei Guo, Yujing Zhang, Wenjia Zhang, Butian Zhang, Xiaoli Song, Jinliang Niu

**Affiliations:** 1 Department of Radiology, Second Hospital of Shanxi Medical University, Taiyuan, Shanxi, China; 2 Clinical AI Imaging Research Center, Department of Medical Imaging, Shanxi Medical University, Taiyuan, Shanxi, China; 3 Department of Radiology, China-Japan Union Hospital of Jilin University, Changchun, Jilin, China

**Keywords:** bone metastasis, multiparametric MRI, nomogram, periprostatic adipose tissue, prostate cancer, radiomics

## Abstract

**Purpose:**

To develop and validate a multiparametric MRI (mpMRI)-based radiomics model incorporating features from both the tumor lesion and periprostatic adipose tissue (PPAT) for predicting synchronous bone metastasis (BM) in patients with newly diagnosed prostate cancer (PCa).

**Methods:**

This retrospective study enrolled 237 patients with histologically confirmed PCa who underwent prostate mpMRI between January 2021 and December 2024. Patients were randomly allocated to a training cohort (n = 165) and a validation cohort (n = 72) in a 7:3 ratio. Univariate and multivariate logistic regression analyses identified independent clinical predictors of BM. Radiomics features were extracted from tumor lesions and PPAT on T2-weighted imaging (T2WI), fat-suppressed T2-weighted imaging (T2WI-FS), and apparent diffusion coefficient (ADC) maps. Separate radiomics models were constructed for intratumoral, PPAT, and combined (intratumoral + PPAT) features. A combined clinical-radiomics model was established by integrating the radiomics score (Rad-score) with independent clinical predictors. Model performance was evaluated using receiver operating characteristic (ROC) curves, calibration curves, and decision curve analysis (DCA).

**Results:**

Multivariate analysis identified clinical T stage (OR = 5.00, 95% CI: 1.65–16.70; p = 0.006) and Ki-67 expression (OR = 4.61, 95% CI: 1.63–14.10; p = 0.005) as independent predictors of BM. The intratumoral radiomics model achieved AUCs of 0.928 (training) and 0.835 (validation). The PPAT radiomics model achieved AUCs of 0.859 (training) and 0.842 (validation). The combined radiomics model (intratumoral + PPAT) yielded AUCs of 0.955 (95% CI: 0.927–0.982) and 0.850 (95% CI: 0.745–0.954) in the training and validation cohorts, respectively. The combined clinical-radiomics model demonstrated AUCs of 0.960 (95% CI: 0.934–0.986) and 0.873 (95% CI: 0.785–0.960), respectively. DCA indicated favorable clinical net benefit across a wide range of threshold probabilities.

**Conclusion:**

The combined model integrating PPAT and tumor radiomics features with clinical predictors demonstrated robust discriminative ability for predicting BM in newly diagnosed PCa. This non-invasive model may provide complementary risk stratification beyond conventional clinical assessment by identifying patients with potentially aggressive disease characteristics and supporting individualized clinical decision-making.

## Introduction

Prostate cancer (PCa) remains one of the most commonly diagnosed malignancies among men and ranks as the fifth leading cause of cancer-related death in males worldwide (1). Bone is the most frequent site of distant metastasis in PCa, occurring in more than 80% of patients with advanced disease (2). Bone metastasis (BM) confers a poor prognosis, with a reported median survival of approximately 24 months (3). Moreover, patients with BM are at substantial risk of skeletal-related events (SREs), including pathological fractures, bone pain, hypercalcemia, and spinal cord compression. SREs affect up to half of all patients with advanced PCa and can severely impair quality of life, mobility, and daily functional status (4). Therefore, early identification of patients at high risk for BM is essential for guiding treatment decisions, optimizing therapeutic strategies, and improving clinical outcomes.

Current approaches for predicting BM risk in PCa primarily rely on clinical parameters such as serum prostate-specific antigen (PSA) level, clinical T stage, Gleason score (GS), and alkaline phosphatase (ALP). However, the predictive accuracy of these individual markers remains inconsistent, and their combined discriminative power is limited (5, 6). The National Comprehensive Cancer Network (NCCN) guidelines recommend bone scintigraphy for patients with PSA ≥ 20 ng/mL, GS ≥ 8, T3–T4 disease, or bone-related symptoms. Nevertheless, these criteria may not completely distinguish patients with different metastatic risks, potentially resulting in suboptimal selection of patients for metastatic evaluation. Recently, prostate-specific membrane antigen positron emission tomography (PSMA-PET) has emerged as an advanced molecular imaging modality for metastatic assessment in patients with high-risk PCa. Compared with conventional imaging modalities, PSMA-PET has demonstrated superior accuracy for detecting pelvic nodal and distant metastatic disease and has increasingly been incorporated into initial staging strategies for high-risk PCa (7). However, the availability and accessibility of PSMA-PET remain variable across different clinical settings. Therefore, there remains an unmet clinical need for objective, accurate, and non-invasive biomarkers that can improve metastatic risk stratification at diagnosis and identify patients who may benefit from intensified staging evaluation.

Multiparametric magnetic resonance imaging (mpMRI) has become integral to PCa management, offering comprehensive anatomical and functional information through T2-weighted imaging (T2WI), diffusion-weighted imaging (DWI), apparent diffusion coefficient (ADC) mapping, and dynamic contrast-enhanced (DCE) sequences (8). However, conventional mpMRI interpretation remains predominantly qualitative and operator-dependent, limiting its capacity to capture the subvisual biological information embedded within both the tumor and its surrounding microenvironment. Handcrafted radiomics, an emerging computational approach that extracts high-throughput quantitative features from medical images, provides a transformative opportunity to overcome these limitations by converting imaging data into mineable features that reflect underlying tissue heterogeneity (9–15). Several prior studies have successfully demonstrated the utility of MRI-based radiomics features derived from intratumoral regions for predicting BM in PCa (16–18).

However, existing radiomics prediction models have predominantly focused on the tumor in isolation, overlooking the biologically influential periprostatic adipose tissue (PPAT). Emerging evidence has highlighted the critical role of PPAT in PCa progression. PPAT may promote tumor growth and metastasis through the secretion of adipokines such as adiponectin, tumor necrosis factor-α (TNF-α), and chemokine (C-C motif) ligand 7 (CCL7), which can subsequently stimulate tumor growth and migration (19, 20). Qualitative radiological assessments of PPAT, including measurements of periprostatic fat thickness, have been established as predictive markers for aggressive disease features, including high GS, lymph node metastasis (LNM), and BM (21–23). More recently, Liu et al. demonstrated that a nomogram integrating the Rad-score of PPAT with alkaline phosphatase (ALP) and clinical N stage (cN) showed excellent performance in predicting BM in newly diagnosed PCa, achieving an AUC of 0.908 (24). However, that study focused on PPAT alone without incorporating intratumoral imaging information, potentially underutilizing the available imaging data.

To date, no study has systematically combined handcrafted radiomics features extracted from both the primary tumor and the surrounding PPAT with clinical predictors for BM prediction. We hypothesized that integrating handcrafted radiomics features from both tissue compartments would yield superior predictive performance compared with either compartment alone, reflecting the complementary biological information captured from the tumor and its microenvironment. Therefore, the aim of this study was to develop and validate a combined prediction model integrating mpMRI-derived radiomics features from both the tumor lesion and PPAT with independent clinical risk factors for individualized prediction of BM in patients with newly diagnosed PCa. Beyond conventional clinical risk stratification, this model was designed to provide additional information for identifying patients with potentially aggressive disease characteristics and improving metastatic risk assessment at diagnosis. By incorporating quantitative imaging features reflecting both tumor heterogeneity and the surrounding microenvironment, this model may serve as a complementary tool to support individualized clinical decision-making, including the identification of patients who may benefit from further diagnostic evaluation or intensified staging strategies.

## Materials and methods

This retrospective study was approved by the Institutional Review Board of the Second Hospital of Shanxi Medical University. The requirement for written informed consent was waived due to the retrospective nature of the study. This retrospective study was evaluated and reported according to the Radiomics Quality Score 2.0 (RQS 2.0) framework (25) and the Transparent Reporting of a multivariable prediction model for Individual Prognosis or Diagnosis (TRIPOD) guidelines. The present study employed handcrafted radiomics based on multiparametric MRI. Relevant methodological domains considered according to the RQS 2.0 framework included clinical objective definition, imaging acquisition, image preprocessing, segmentation strategy, radiomics feature extraction, feature reproducibility assessment, feature selection, model development, internal validation, and clinical utility evaluation. Methodological assessment of the present study according to relevant domains of the Radiomics Quality Score 2.0 (RQS 2.0) is summarized in **Supplementary Table 1**.

### Patient selection

**Figure 1:**
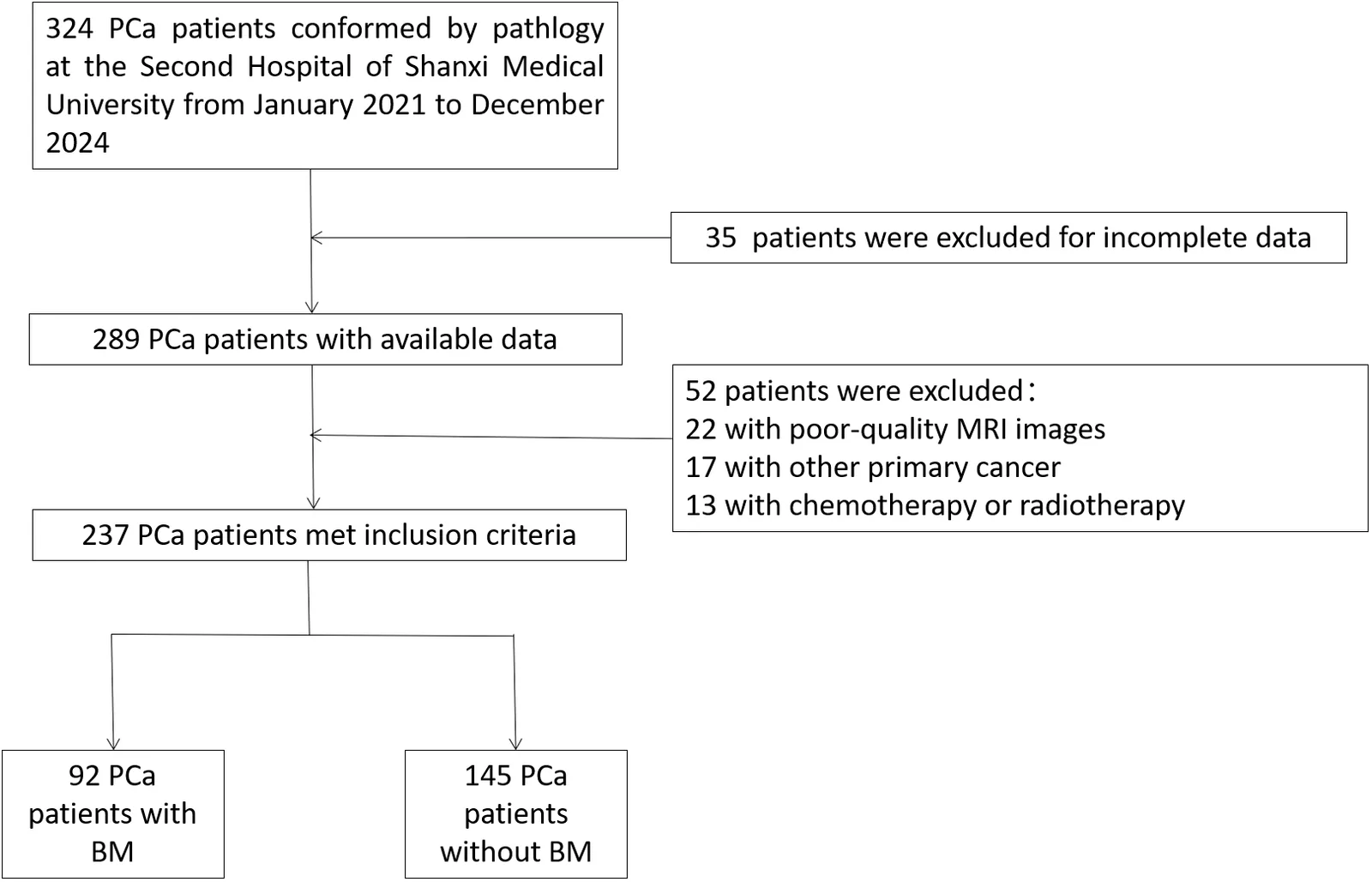
The flow chart for the exclusion of patients. PCa, prostate cancer; BM, bone metastasis.

Consecutive patients diagnosed with PCa at the Second Hospital of Shanxi Medical University between January 2021 and December 2024 were retrospectively screened. The inclusion criteria were as follows: (a) histologically confirmed primary PCa via radical prostatectomy or systematic 12-core transrectal/transperineal biopsy (MRI-ultrasound fusion targeted biopsy was not available at our institution during the study period); (b) prostate mpMRI performed within 30 days before or after the date of histological diagnosis and prior to any anticancer treatment; (c) definitive determination of BM status by emission computed tomography (ECT) bone scintigraphy and/or PET-CT; and (d) availability of complete clinical and pathological data. The exclusion criteria were as follows: (a) incomplete or inadequate clinical information; (b) MRI images of insufficient quality for reliable segmentation (e.g., significant motion artifacts or incomplete coverage of the prostate); (c) receipt of any anticancer therapy (e.g., chemotherapy, radiotherapy, or hormonal therapy) prior to the prostate MRI examination; (d) history of, or concurrent, other primary malignancies; and (e) suspicious bone lesions that remained equivocal on both ECT and PET-CT. The patient enrollment process is illustrated in **Figure 1**.

### Clinical data collection

Clinical and pathological data were collected from electronic medical records, including age, body mass index (BMI), preoperative total PSA (t-PSA) level, clinical T stage (cT) according to the American Joint Committee on Cancer (AJCC) TNM staging system (8th edition), clinical lymph node status (cN), biopsy GS (categorized as < 7 vs. ≥ 7), Ki-67 expression level (Ki-67 expression level was determined from pretreatment prostate biopsy specimens using immunohistochemical staining, dichotomized at a 10% cutoff based on institutional standard), and Prostate Imaging-Reporting and Data System (PI-RADS v2.1) score.

BM status was determined by ECT bone scintigraphy and/or PET-CT at the time of initial diagnosis, within 30 days of the prostate mpMRI and prior to any anticancer treatment. BM was characterized by multiple foci of increased radionuclide uptake predominantly involving the axial skeleton, with occasional areas of photopenia. All imaging interpretations were performed by experienced nuclear medicine physicians blinded to the radiomics analysis.

### MRI acquisition protocol

All MRI examinations were performed on a 3.0-T scanner (Discovery MR750W; GE Healthcare, Milwaukee, WI, USA) with a dedicated eight-channel phased-array abdominal surface coil. The imaging protocol, consistent with PI-RADS v2.1 recommendations, included the following sequences:

1. Axial T2WI: field of view (FOV) = 200 × 200 mm²; repetition time (TR)/echo time (TE) = 4250/98 ms; slice thickness = 3.5 mm; slice gap = 0.5 mm.
2. Axial T2WI with fat suppression (T2WI-FS): FOV = 200 × 200 mm²; TR/TE = 3500/90 ms; slice thickness = 3.5 mm; slice gap = 0.5 mm.
3. Axial DWI: FOV = 220 × 220 mm²; TR/TE = 4800/86 ms; slice thickness = 3.5 mm; slice gap = 0.5 mm; b-values = 0 and 1400 s/mm².

ADC maps were automatically generated from the DWI data using the scanner’s built-in software.

### Lesion segmentation

Regions of interest (ROIs) for both the tumor lesion and PPAT were manually delineated using the open-source software ITK-SNAP (version 3.8.0; www.itksnap.org) on axial T2WI, T2WI-FS, and ADC map images. All segmentations were performed by a genitourinary radiologist (Reader 1) with 5 years of experience in prostate MRI interpretation, who was blinded to BM status and all pathological results.

### PPAT segmentation

The PPAT ROI was delineated on each axial slice according to the following anatomical boundaries: (1) lateral: the endopelvic fascia adjacent to the levator ani muscle; (2) posterior: Denonvilliers’ fascia; (3) anterior: the pubic symphysis; (4) superior: the level of the seminal vesicle base; and (5) inferior: the prostatic apex. Fat tissue within these boundaries was segmented based on signal intensity characteristics on T2WI and T2WI-FS, with care taken to exclude the prostate gland, neurovascular bundles, and pelvic vessels.

### Tumor segmentation

The tumor ROI was segmented slice-by-slice along the outermost margins of the lesion as identified on T2WI, with DWI/ADC used for cross-referencing. Structures including the urethra, ejaculatory ducts, verumontanum, and the roots of the seminal vesicles were carefully excluded. In cases of multifocal disease, only the dominant (largest) lesion was annotated, consistent with the PI-RADS index lesion concept.

**Figure 2:**
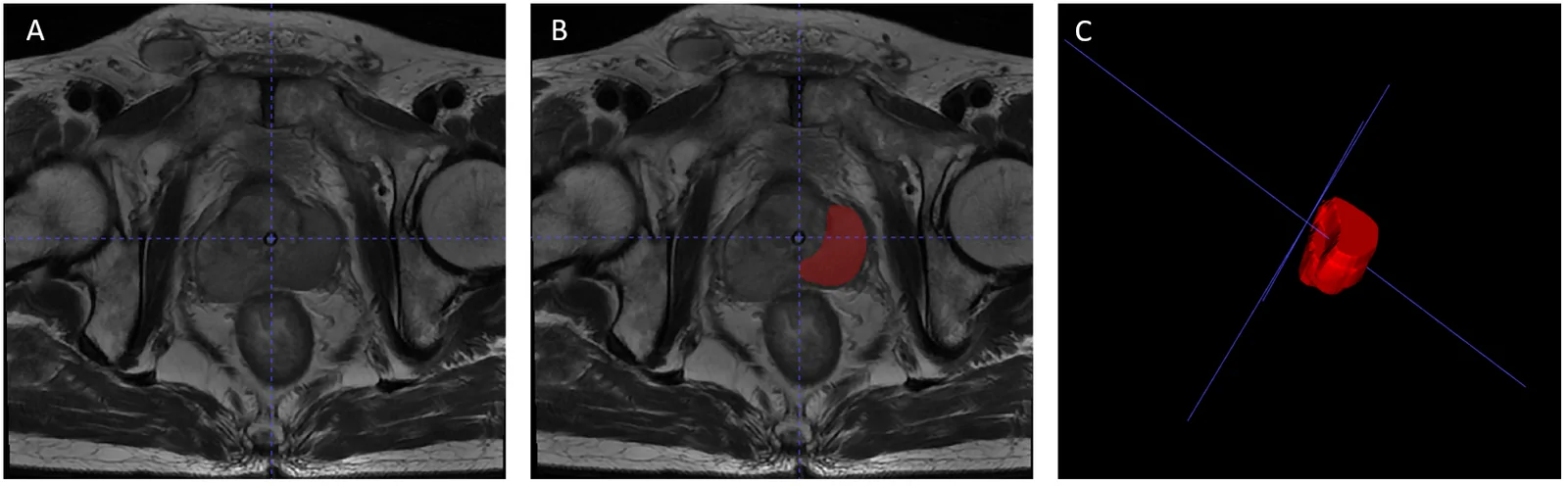
Schematic diagram of ROI for the tumor lesion in MRI images of prostate cancer patients. **(A)** T2WI sequence showing prostate cancer in the intratumoral region. **(B)** Outline of the intratumoral ROI in the T2WI sequence. **(C)** Generated intratumoral ROI in the T2WI sequence. MRI, magnetic resonance imaging; ROI, regions of interest; T2WI, T2-weighted imaging.

**Figure 3:**
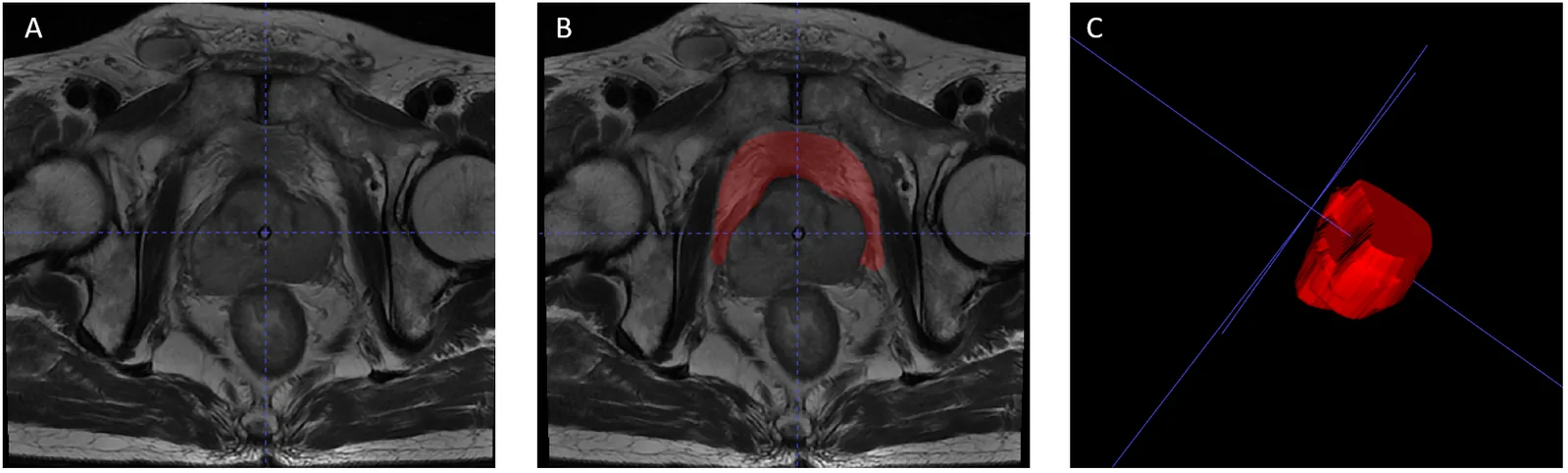
Schematic diagram of ROI for the periprostatic adipose tissue (PPAT) in MRI images of prostate cancer patients. **(A)** T2WI sequence showing prostate cancer in the PPAT region. **(B)** Outline of the PPAT ROI in the T2WI sequence. **(C)** Generated PPAT ROI in the T2WI sequence. MRI, magnetic resonance imaging; PCa, prostate cancer; PPAT, periprostatic adipose tissue; ROI, regions of interest; T2WI, T2-weighted imaging.

All segmentations were subsequently independently reviewed and confirmed by a senior genitourinary radiologist (Reader 2) with 10 years of experience. In cases of disagreement, consensus was reached through joint discussion. The software then generated the three-dimensional volume of interest (VOI) for each tissue region. Representative ROIs for both tumor lesions and PPAT are illustrated in **Figures 2** and **3**.

### Reproducibility assessment

To evaluate intra-observer reproducibility, Reader 1 independently re-segmented 30 randomly selected cases after a minimum interval of one month, blinded to the initial segmentation results. The intraclass correlation coefficient (ICC) was calculated for all extracted radiomics features; features with ICC ≥ 0.75 were considered to have good reproducibility and were retained for subsequent analysis.

### Radiomics feature extraction

Radiomics features were extracted using FeAture Explorer (FAE, version 0.5.8) implemented in Python. Prior to feature extraction, all images were resampled to an isotropic voxel size of 1.0 × 1.0 × 1.0 mm³ using B-spline interpolation, and image intensities were normalized using z-score normalization. Gray-level intensities were discretized into 80 bins, consistent with prior radiomics studies (26). The following seven categories of features were extracted from each ROI on each MRI sequence: (1) shape features; (2) first-order statistical features; (3) gray-level co-occurrence matrix (GLCM) features; (4) gray-level run-length matrix (GLRLM) features; (5) gray-level dependence matrix (GLDM) features; (6) gray-level size-zone matrix (GLSZM) features; and (7) neighboring gray-tone difference matrix (NGTDM) features. A total of 1,688 features were extracted per ROI per MRI sequence, yielding 10,128 initial features across all six ROI–sequence combinations (2 tissue types × 3 sequences).

### Radiomics feature selection and model construction

All feature selection and model construction procedures were performed exclusively on the training cohort. The multi-step feature selection pipeline was as follows:

Step 1 — Reproducibility filtering: Features with intra-observer ICC ≥ 0.75 were retained to ensure measurement stability.

Step 2 — Z-score normalization: Retained features were standardized to zero mean and unit variance to eliminate the effects of differing scales.

Step 3 — Univariate screening: The normality of each feature was assessed using the Shapiro-Wilk test. Normally distributed features were compared between BM and non-BM groups using independent-sample t-tests; non-normally distributed features were compared using Mann–Whitney U tests. Features with p < 0.05 were retained.

Step 4 — Collinearity reduction: The Spearman rank correlation matrix was computed; for each pair of features with |ρ| > 0.9, the feature with lower univariate discriminative ability was removed.

Step 5 — LASSO regression: Least absolute shrinkage and selection operator (LASSO) logistic regression with 10-fold cross-validation was applied to select the optimal subset of features for each single-sequence model. The regularization parameter λ was chosen at the minimum cross-validated deviance.

Step 6 — Multi-sequence integration: Bidirectional stepwise logistic regression based on the Akaike information criterion (AIC) was applied to select optimal features from the three single-sequence models, constructing separate multi-sequence radiomics models for the PPAT and intratumoral compartments.

Step 7 — Multi-tissue integration: Bidirectional stepwise logistic regression was used to combine the PPAT and intratumoral multi-sequence models into a unified radiomics model, and a radiomics score (Rad-score) was computed as the linear predictor for each patient.

### Clinical model development

Univariate logistic regression was performed to evaluate the association of each clinical variable (age, BMI, t-PSA, cT stage, cN status, GS, PI-RADS score, and Ki-67 expression) with BM status in the training cohort. Variables with p < 0.05 in univariate analysis were entered into a multivariate logistic regression model using backward elimination. Variables retaining statistical significance (p < 0.05) in the multivariate analysis were considered independent predictors and were used to construct the clinical prediction model.

### Combined model and nomogram development

The independent clinical predictors were combined with the Rad-score in a multivariate logistic regression model to construct the combined clinical-radiomics model. A nomogram was generated to visualize the combined model and facilitate clinical application. An illustrative case example of nomogram-based risk estimation is presented alongside the nomogram.

### Model evaluation and comparison

Discriminative performance was evaluated using the area under the ROC curve (AUC) with 95% confidence intervals (CIs). AUC comparisons between models were performed using the DeLong test. Calibration was assessed using the Hosmer-Lemeshow (H-L) goodness-of-fit test and calibration curves generated from 1,000 bootstrap resamples. Clinical utility was evaluated by DCA, which plots net benefit against a range of threshold probabilities. Sensitivity (SEN), specificity (SPE), accuracy (ACC), positive predictive value (PPV), and negative predictive value (NPV) were calculated at the optimal cutoff determined by the Youden index. Confidence intervals for these metrics were estimated using 2,000 bootstrap resamples.

### Statistical analysis

Statistical analyses were performed using R software (version 4.2.3; R Foundation for Statistical Computing, Vienna, Austria) with the “glmnet,” “rms,” “pROC,” “rmda,” and “ResourceSelection” packages, and IBM SPSS Statistics (version 25.0; IBM Corp., Armonk, NY, USA). Continuous variables were presented as median [interquartile range (IQR)] for non-normally distributed data or mean ± standard deviation (SD) for normally distributed data. Comparisons between groups were performed using the Mann–Whitney U test or independent-sample t-test, as appropriate. Categorical variables were expressed as frequencies (percentages) and compared using the chi-square test or Fisher’s exact test. All statistical tests were two-sided, and a p-value < 0.05 was considered statistically significant.

## Results

### Patient characteristics

A total of 237 patients (median age 72 years; IQR 67–78) were included in the final analysis, comprising 92 patients (38.8%) with BM and 145 patients (61.2%) without BM. All BM patients in this study were synchronous, detected at the time of initial diagnosis by ECT bone scintigraphy and/or PET-CT performed within 30 days of the prostate mpMRI. Patients with BM at diagnosis generally received systemic therapy. Patients were randomly allocated to the training and validation cohorts in a 7:3 ratio using stratified random sampling based on bone metastasis (BM) status to ensure balanced representation of BM-positive and BM-negative cases across both cohorts. The training cohort comprised 165 patients [70 with BM (42.4%)], and the validation cohort comprised 72 patients [22 with BM (30.6%)]. There were no statistically significant differences between the training and validation cohorts with respect to age, BMI, t-PSA, BM status, cT stage, cN status, GS, PI-RADS score, or Ki-67 expression (all p > 0.05), as detailed in **Table 1**.

**Table 1:** Baseline characteristics of patients.

Characteristic	Training group (165)	Validation group (72)	P-value
Age [years-median (IQR)]	71 (66,77.5)	73 (68.25,79.75)	0.118
BMI (kg/m^2^mean ± SD)	23.14 ± 3.31	24.01 ± 3.43	0.872
t-PSA [ng/mL-median (IQR)]	88.18 (23.11,245.96)	81.42 (28.85,239.87)	0.947
BM [n-(%)]			0.085
no	95 (57.6%)	50 (69.4%)	
yes	70 (42.4%)	22 (30.6%)	
cT [n-(%)]			0.514
T1-T2	74 (44.8%)	29 (40.3%)	
T3-T4	91 (55.2%)	43 (59.7%)	
cN [n-(%)]			0.591
LN-negative	118 (71.5%)	49 (68.1%)	
LN-positive	47 (28.5%)	23 (31.9%)	
GS score [n-(%)]			0.391
<7	20 (12.1%)	6 (11%)	
≥7	145 (87.9%)	66 (89%)	
PI-RADS [n-(%)]			0.328
≤3	12 (7.3%)	8 (11.1%)	
≥4	153 (92.7%)	64 (88.9%)	
Ki67 [n-(%)]			0.494
<10%	70 (42.4%)	34 (47.2%)	
≥10%	95 (57.6%)	38 (52.8%)	

BMI, Body Mass Index; t-PSA, Total Prostate-specific Antigen; BM, Bone Metastasis; cT, Clinical T stage; cN, Clinical N stage; GS, Gleason score; PI-RADS, Prostate Imaging Reporting and Data System. *Significance at p< 0.050.

### Independent clinical predictors of bone metastasis

Univariate logistic regression analysis identified t-PSA (OR = 1.002, p < 0.001), cT stage (OR = 17.64, p < 0.001), cN status (OR = 8.09, p < 0.001), GS (OR = 7.95, p < 0.001), PI-RADS score (OR = 10.56, p < 0.001), and Ki-67 expression (OR = 12.16, p < 0.001) as significant univariate predictors of BM, whereas age and BMI were not statistically significant (**Table 2**). In the multivariate logistic regression model, only two variables retained independent statistical significance: cT stage (OR = 5.00, 95% CI: 1.65–16.70; p = 0.006) and Ki-67 expression (OR = 4.61, 95% CI: 1.63–14.10; p = 0.005). These two variables were used to construct the clinical prediction model.

**Table 2:** The clinical factors analysis in the training cohort.

Characteristic	Univariate analysis		Multivariate analysis	
	OR (95% CI)	P-value	OR (95% CI)	P-value
Age	0.995 (0.960 - 1.032)	0.955		
BMI	0.957 (0.870 - 1.051)	0.355		
t-PSA	1.002 (1.001 - 1.003)	< 0.001*	1.00 (0.999-1.001)	0.068
T stage	17.64 (7.86 - 44.26)	< 0.001*	5.00 (1.65-16.70)	0.006*
LN status	8.09 (3.80 - 18.40)	< 0.001*	1.55 (0.58-4.18)	0.383
GS score	7.95 (2.19 - 51.16)	< 0.001*	0.55 (0.09-4.46)	0.531
PI-RADS score	10.56 (3.83 - 44.20)	< 0.001*	1.68 (0.51-7.93)	0.442
Ki67 expression	12.16 (5.60 - 29.04)	< 0.001*	4.61 (1.63-14.10)	0.005*

OR, Odds ratio; CI, confidence interval; BMI, Body Mass Index; t-PSA, Total Prostate-specific Antigen; BM, Bone Metastasis; cT, Clinical T stage; cN, Clinical N stage; GS, Gleason score; PI-RADS, Prostate Imaging Reporting and Data System. *Significance at p< 0.050.

The clinical model based on cT stage and Ki-67 expression achieved an AUC of 0.835 (95% CI: 0.778–0.892) in the training cohort and 0.777 (95% CI: 0.678–0.875) in the validation cohort (**Table 3**).

**Table 3:** Predictive performance of each model in the training and validation cohorts.

Model	Set	AUC (95% CI)	SEN	SPE	ACC
PPAT radiomics	Training	0.859 (0.802-0.914)	0.746	0.861	0.812
	Validation	0.842 (0.729-0.954)	0.863	0.740	0.777
Intratumoral radiomics	Training	0.928 (0.890–0.963)	0.873	0.840	0.854
	Validation	0.835 (0.724-0.946)	0.909	0.540	0.652
clinical	Training	0.835(0.778–0.892)	0.800	0.789	0.793
	Validation	0.777(0.678–0.875)	0.636	0.760	0.722
radiomics	Training	0.955(0.927–0.982)	0.914	0.894	0.903
	Validation	0.850(0.745–0.954)	0.909	0.560	0.666
Nomogram	Training	0.960(0.934–0.986)	0.985	0.831	0.897
	Validation	0.872(0.785–0.960)	0.954	0.460	0.611

AUC, area under the curve; CI, confidence interval; SEN, Sensitivity; SPE, Specificity; ACC, Accuracy.

### Radiomics feature selection and model performance

From the initial pool of 1,688 features per ROI per sequence, the multi-step feature selection pipeline yielded the following retained features for single-sequence models: 3, 6, and 2 intratumoral features from T2WI, T2WI-FS, and ADC sequences, respectively, and 2, 4, and 1 PPAT features from T2WI, T2WI-FS, and ADC sequences, respectively. After multi-sequence integration via bidirectional stepwise regression, 9 intratumoral features and 4 PPAT features were retained. Following the final multi-tissue integration step, 13 features were included in the unified radiomics model (**Supplementary Table 2**).

The performance of individual and combined radiomics models was as follows:

PPAT radiomics model: AUC of 0.859 (95% CI: 0.802–0.914) in the training cohort and 0.842 (95% CI: 0.729–0.954) in the validation cohort.

Intratumoral radiomics model: AUC of 0.928 (95% CI: 0.890–0.963) in the training cohort and 0.835 (95% CI: 0.724–0.946) in the validation cohort.

Combined radiomics model (intratumoral + PPAT): AUC of 0.955 (95% CI: 0.927–0.982) in the training cohort and 0.850 (95% CI: 0.745–0.954) in the validation cohort.

**Figure 4:**
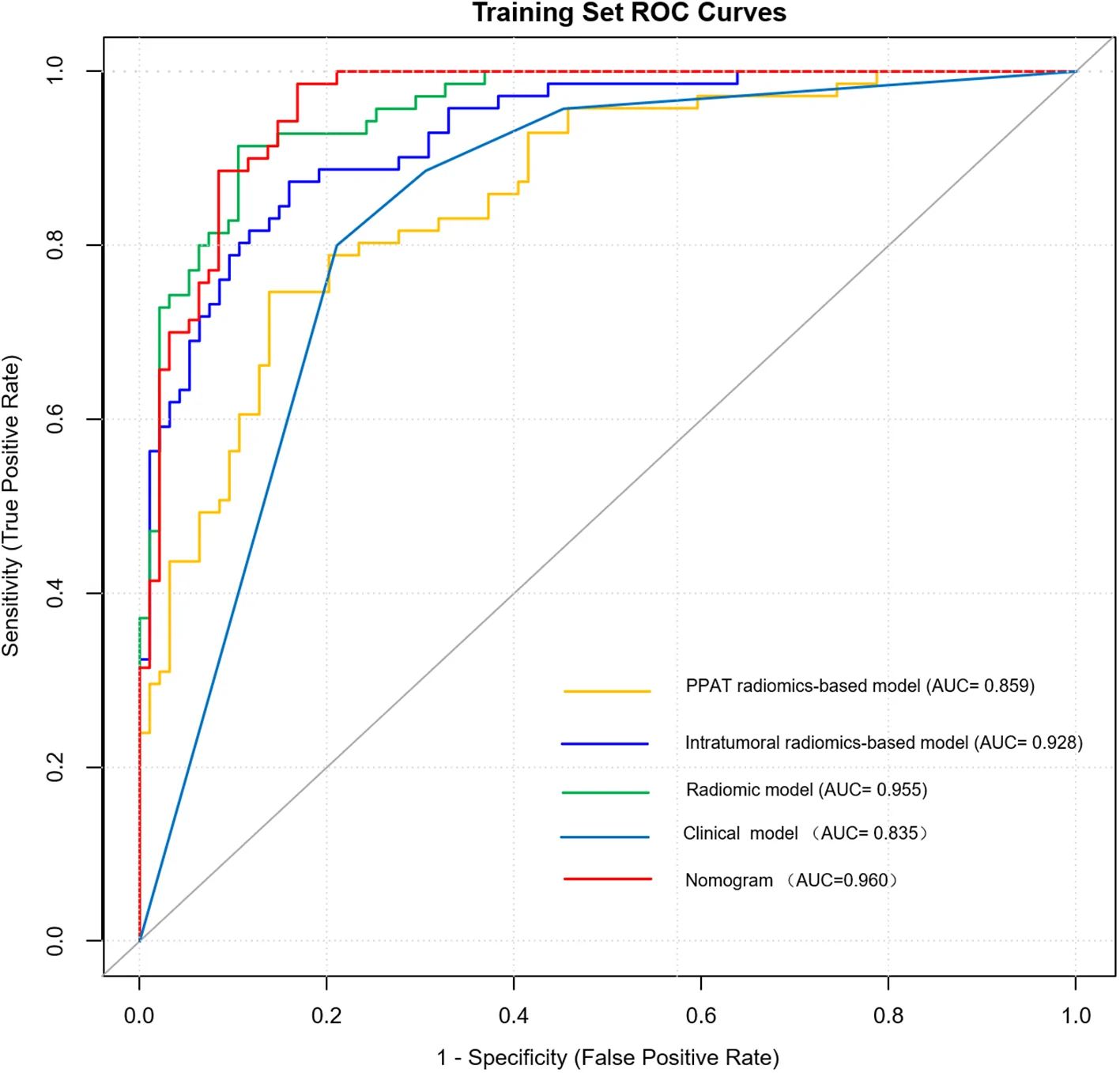
ROC curves for different models in the training group. The Receiver Operating Characteristic (ROC) curves of the five models for training cohort can be seen in the panel.

**Figure 5:**
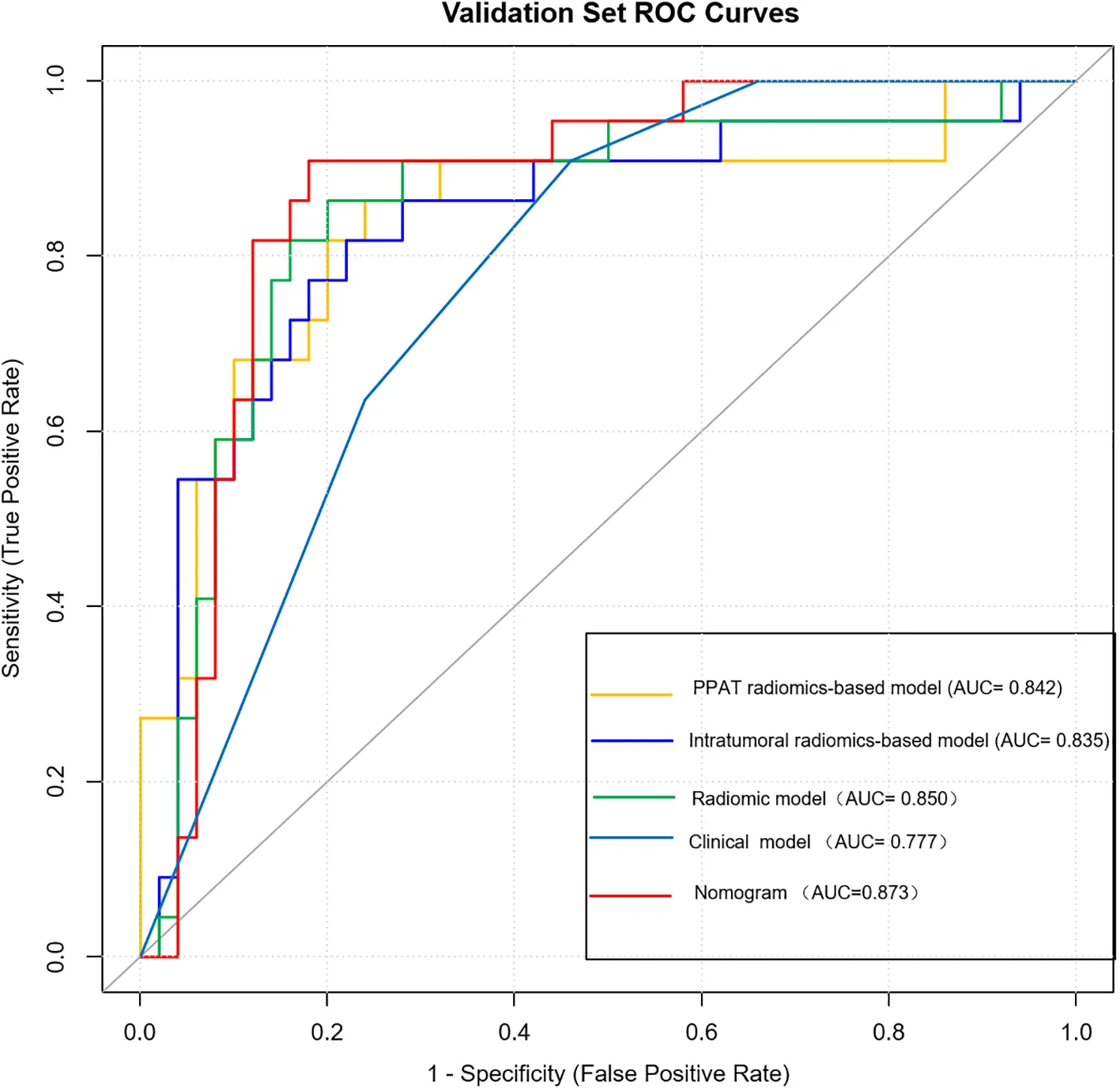
ROC curves for different models in the validation group. The Receiver Operating Characteristic (ROC) curves of the five models for validation cohort can be seen in the panel.

While the intratumoral radiomics model outperformed the PPAT model in the training cohort, the PPAT model demonstrated comparable validation performance (AUC 0.842 vs. 0.835), with notably lower AUC degradation between training and validation (Δ = 0.017 vs. Δ = 0.093). The combined radiomics model incorporating both tissue compartments achieved the highest training AUC (0.955) with an acceptable validation AUC (0.850), suggesting that PPAT features provide complementary information to intratumoral features. ROC curves are presented in **Figures 4**, **5**, and detailed performance metrics are provided in **Table 3**.

### Combined clinical-radiomics model (nomogram)

**Figure 6:**
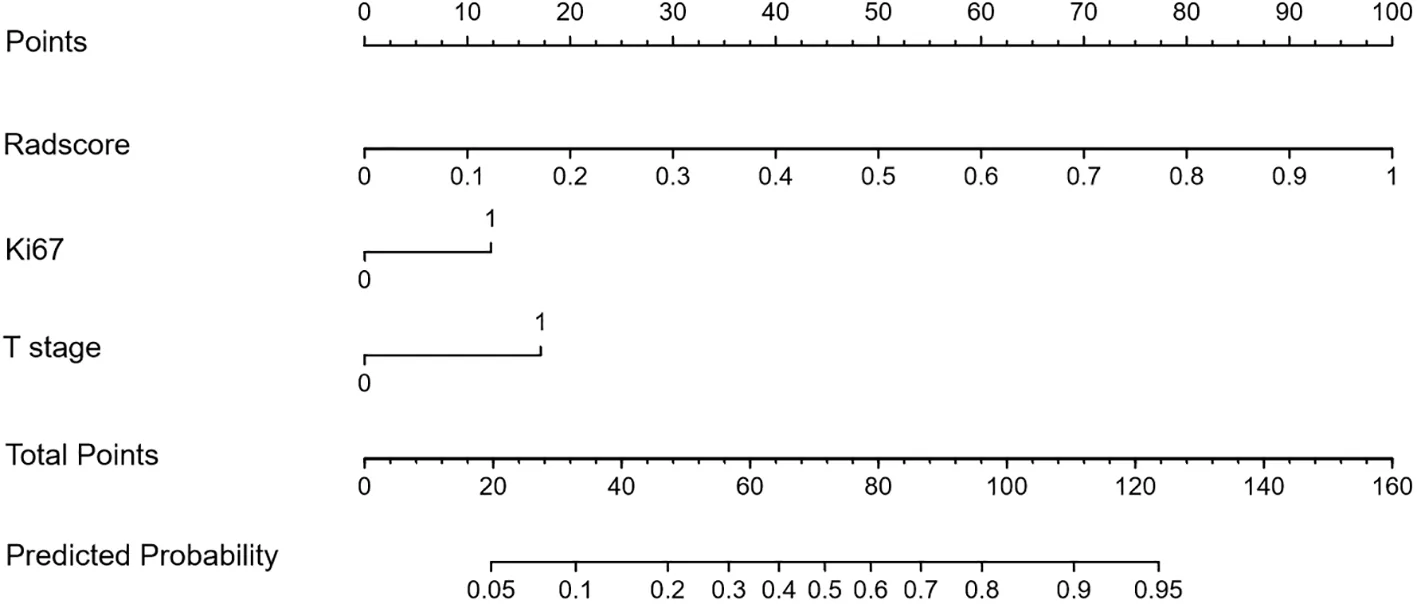
Radiomics nomogram. The Radiomics Nomogram is the prediction model based on radiomics that has been developed to estimate the likelihood of bone metastasis in prostate cancer (PCa) patients using the training dataset.

**Figure 7:**
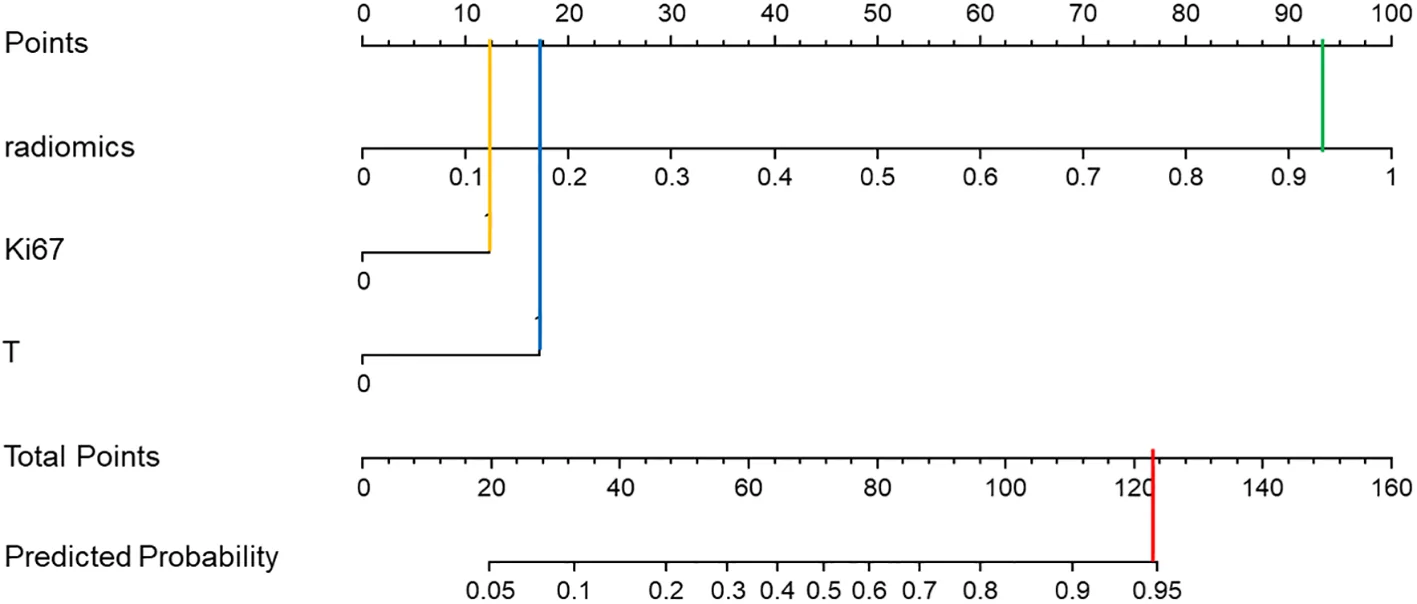
To utilize this model, follow these steps: (1) Identify the patient’s radiomics score, Ki67 expression, T stage, then draw a vertical line for each corresponding axis to determine their individual scores; (2) Add up these scores; (3) Locate the total score on the cumulative points axis and draw a descending line to evaluate the patient’s risk of bone metastasis. (12.5 + 17.5 + 93 = 123).

**Figure 8:**
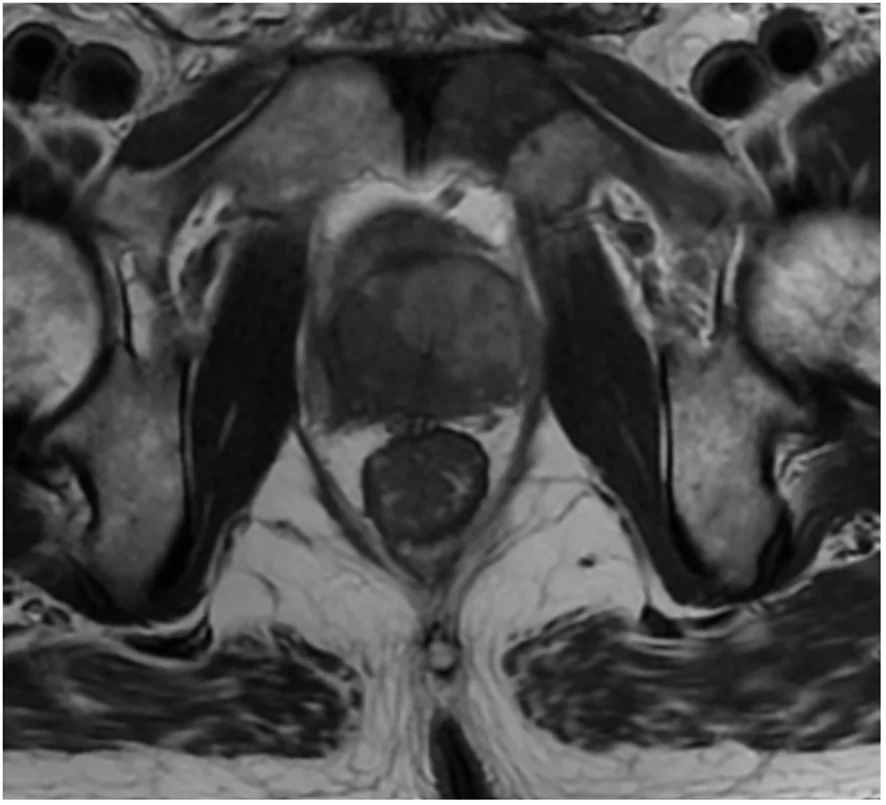
Prostate mpMRI with T2WI was performed in a 67-year-old man with bone metastasis. MRI, magnetic resonance imaging; T2WI, T2-weighted imaging.

**Figure 9:**
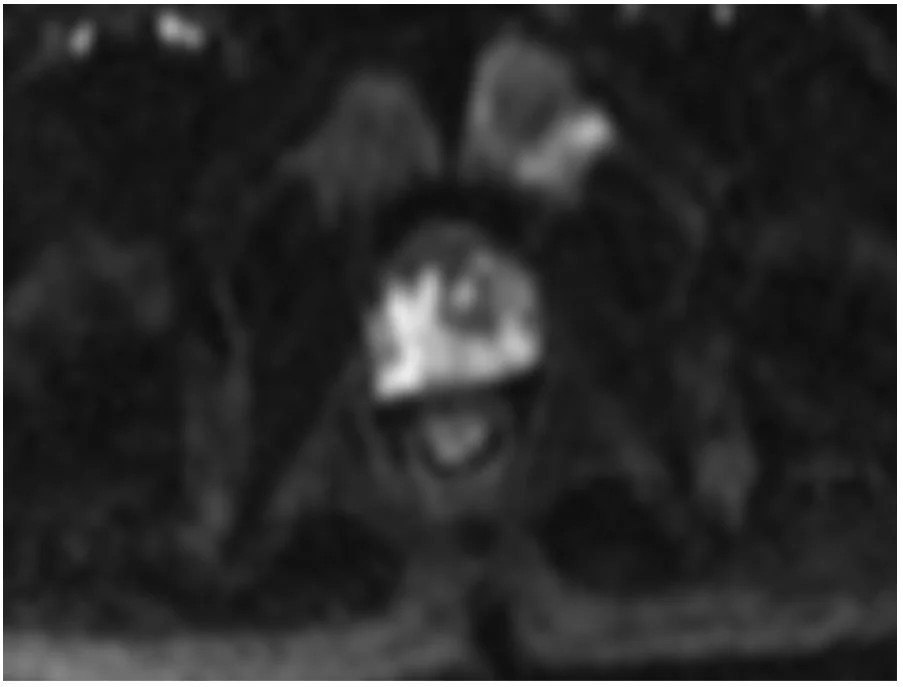
Prostate mpMRI with DWI was performed in a 67-year-old man with bone metastasis. MRI, magnetic resonance imaging; DWI, diffusion-weighted imaging.

The combined model integrated cT stage, Ki-67 expression, and the Rad-score via multivariate logistic regression. The nomogram is presented in **Figure 6**. An illustrative case demonstrating the clinical application of the model is shown in **Figures 7**–**9**. To investigate the biological relevance of MRI radiomic features, we additionally evaluated their associations with Ki-67 expression status. Among the 13 selected radiomic features extracted from intratumoral regions and periprostatic adipose tissue (PPAT), 12 features showed significant differences between Ki-67-negative and Ki-67-positive groups after FDR correction (FDR <0.05). These findings indicate that MRI-derived radiomic features may capture imaging characteristics associated with tumor proliferative activity. Detailed results are summarized in **Supplementary Table 3**.

### Training cohort performance

**Figure 10:**
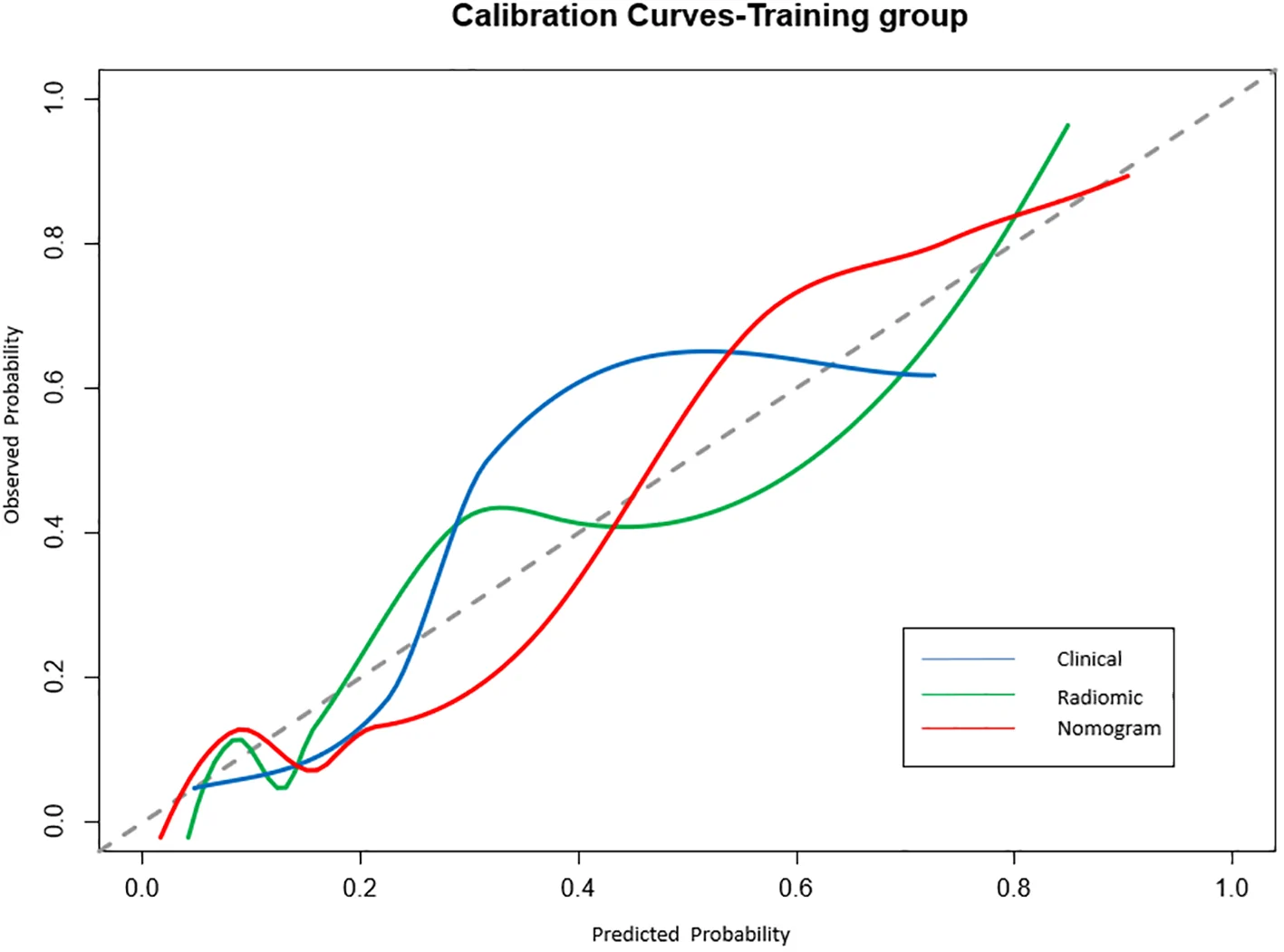
Hosmer-Lemeshow goodness-of-fit test results in the training cohort: clinical (P = 0.928), radiomic (P = 0.727) and nomogram model (P = 0.680).

**Figure 11:**
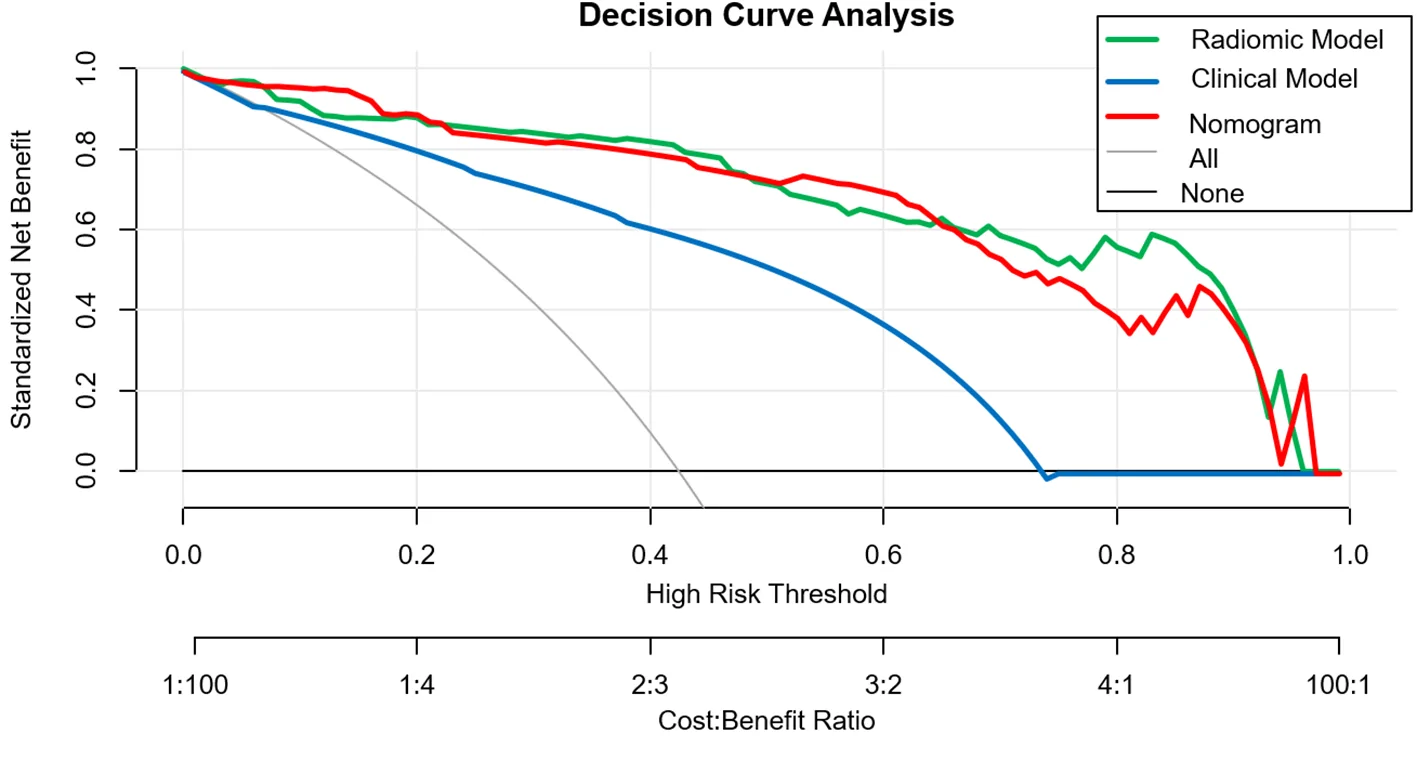
Decision curve analysis (DCA) of the clinical, radiomics, and nomogram models in the training cohort.

The nomogram demonstrated strong predictive performance, achieving an AUC of 0.960 (95% CI: 0.934–0.986). This was significantly higher than the clinical model (AUC = 0.835; DeLong test, p < 0.001) but not significantly different from the combined radiomics model (AUC = 0.955; DeLong test, p = 0.350). The ACC, SEN, and SPE of the nomogram in the training cohort were 0.897, 0.985, and 0.831, respectively. The Hosmer-Lemeshow test indicated adequate calibration (p = 0.680; **Figure 10**). DCA confirmed that the nomogram provided superior net benefit across a wide range of threshold probabilities (**Figure 11**).

### Validation cohort performance

**Figure 12:**
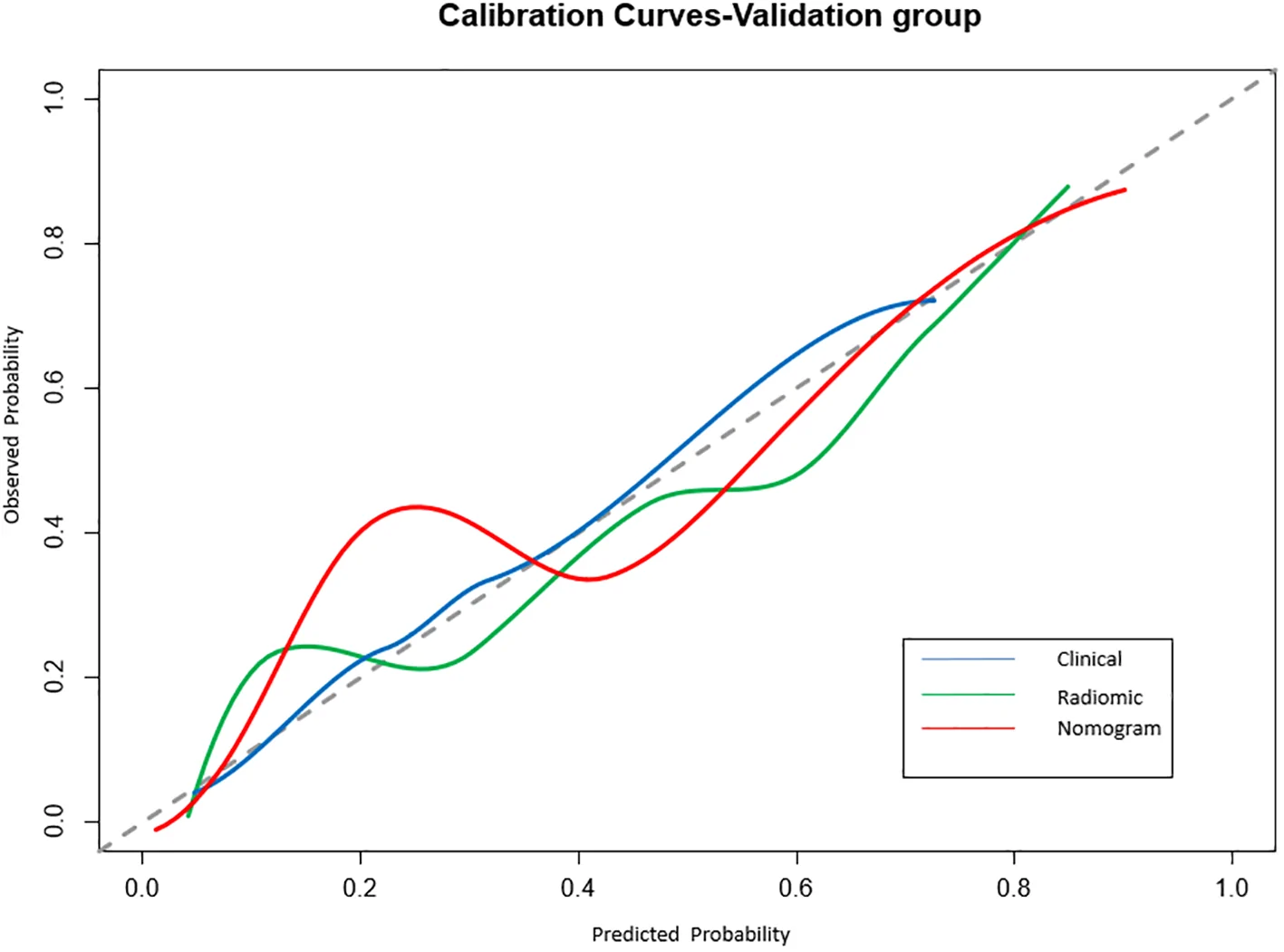
Hosmer-Lemeshow goodness-of-fit test results in the validation cohort: clinical (P = 0.068), radiomic (P = 0.763) and nomogram model (P = 0.706).

**Figure 13:**
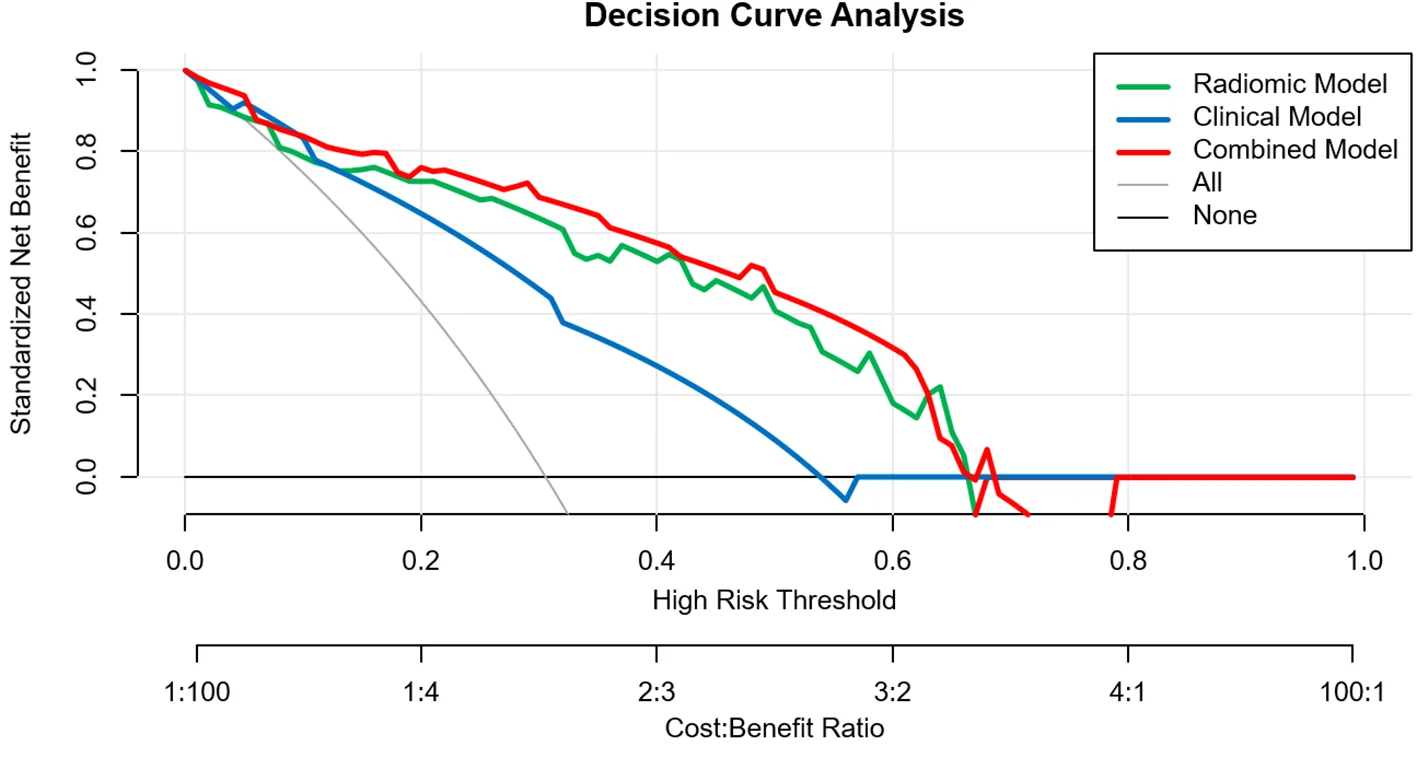
Decision curve analysis (DCA) of the clinical, radiomics, and nomogram models in the validation cohort.

The nomogram maintained high discriminative ability in the validation cohort, with an AUC of 0.873 (95% CI: 0.785–0.960). Although the AUC was numerically higher than both the clinical model (AUC = 0.777) and the combined radiomics model (AUC = 0.850), these differences did not reach statistical significance (DeLong test: p = 0.085 and p = 0.288, respectively). The nomogram demonstrated high sensitivity (SEN = 0.954), indicating its ability to identify the vast majority of patients with BM. However, this was accompanied by moderate specificity (SPE = 0.460) and an overall accuracy of 0.611. All models showed adequate calibration in the validation cohort (Hosmer-Lemeshow test, p > 0.05; **Figure 12**), and DCA consistently demonstrated the favorable clinical net benefit of the nomogram (**Figure 13**).

### Comparison between models

In the training cohort, the DeLong test revealed that the nomogram (AUC = 0.960) was significantly superior to the clinical model (AUC = 0.835; p < 0.001) but was not significantly different from the combined radiomics model (AUC = 0.955; p = 0.350). In the validation cohort, the nomogram (AUC = 0.873) was numerically higher than both the clinical model (AUC = 0.777; p = 0.085) and the combined radiomics model (AUC = 0.850; p = 0.288), although these differences did not reach statistical significance.

These findings indicate that the radiomics features — rather than the clinical variables — constitute the primary driving force behind the model’s predictive power. The addition of clinical T stage and Ki-67 expression to the Rad-score conferred a modest, non-significant improvement in discriminative ability (ΔAUC = 0.005 in training and 0.023 in validation), suggesting that the clinical variables provided limited incremental information beyond what was already captured by the imaging-derived features.

### Calibration and clinical utility

The Hosmer-Lemeshow goodness-of-fit test indicated adequate calibration for all three models in both cohorts: clinical model (training p = 0.928; validation p = 0.068), combined radiomics model (training p = 0.727; validation p = 0.763), and nomogram (training p = 0.680; validation p = 0.706). Calibration curves confirmed reasonable agreement between predicted probabilities and observed outcomes (**Figures 10**, **12**).

DCA demonstrated that the nomogram provided the greatest net clinical benefit across a wide range of threshold probabilities in both the training cohort (**Figure 11**) and the validation cohort (**Figure 13**), consistently outperforming the treat-all and treat-none strategies as well as the clinical model alone.

## Discussion

In this study, we developed and validated a novel combined prediction model that integrates mpMRI-based radiomics features from both the prostate tumor and PPAT with independent clinical risk factors for the individualized prediction of BM in patients with newly diagnosed PCa. To our knowledge, this is the first study to systematically combine quantitative radiomics features from these two anatomically and biologically complementary tissue compartments for BM prediction. The combined clinical-radiomics model demonstrated promising discriminative performance, achieving AUCs of 0.960 and 0.873 in the training and validation cohorts, respectively, and the nomogram provided favorable net clinical benefit across a wide range of threshold probabilities on DCA.

### Radiomics in prostate cancer: context and comparison

MRI-based radiomics prediction models have been widely investigated in various aspects of prostate cancer management, including diagnosis (27), PI-RADS classification (10), extraprostatic extension detection (28), biochemical recurrence prediction (29), and treatment response assessment (30). In the specific domain of BM prediction, several studies have demonstrated promising results. Wang et al. (16) established a combined clinical-radiomics prediction model based on prostate tumor features to predict BM in PCa, achieving an AUC of 0.916 for the combined model, 0.731 for the clinical model, and 0.898 for the radiomics model. Zhang et al. (17) developed a radiomics nomogram incorporating a multiparametric MRI-derived radiomics signature with independent clinical risk factors, reporting AUCs of 0.93 and 0.92 in the training and validation cohorts, respectively. More recently, Zhang et al. (18) constructed prediction models based on deep transfer learning and pathomics features, achieving AUCs of 0.89 and 0.85, respectively, demonstrating that multimodal approaches can serve as valuable predictors of BM risk. However, all of these studies focused exclusively on intratumoral imaging features, without incorporating information from the surrounding periprostatic tissue microenvironment.

### The rationale for integrating PPAT radiomics

With accumulating evidence, an increasing number of studies have demonstrated that PPAT plays an important role in the occurrence, development, and prognosis of prostate cancer (19–23). At a mechanistic level, prostate cancer cells can derive nutrients and energy from periprostatic adipocytes, promoting tumor growth and facilitating metastatic dissemination (31, 32). Altuna-Coy et al. (33) reported that lipid metabolism disturbances in PPAT can influence tumor cell processes by inducing metabolic reprogramming. These molecular interactions provide a biological rationale for the hypothesis that radiomics features extracted from PPAT may encode information relevant to disease aggressiveness and metastatic potential that is not captured by intratumoral features alone.

Several recent studies have begun to explore the radiomics potential of PPAT. Wu et al. (34) demonstrated that MRI-based radiomic features derived from PPAT serve as independent predictors of positive pathological response following neoadjuvant chemohormonal therapy in patients with high-risk non-metastatic PCa. Liu et al. (35) showed that a combined model incorporating PPAT and tumor radiomics with clinical data demonstrated excellent performance in predicting GS upgrading from biopsy to radical prostatectomy (AUC = 0.937, 95% CI: 0.891–0.983). Furthermore, Liu et al. (24) demonstrated that a nomogram integrating the PPAT Rad-score with alkaline phosphatase and clinical N stage showed strong performance in predicting BM (AUC = 0.908, 95% CI: 0.851–0.966). However, that study focused exclusively on PPAT without incorporating intratumoral imaging information, potentially underutilizing the available imaging data.

### Novel integration of dual-compartment radiomics

The present study addresses this gap by comprehensively extracting and combining radiomics features from both the primary tumor and the surrounding PPAT across three mpMRI sequences (T2WI, T2WI-FS, and ADC). Our results support the complementary value of integrating both tissue compartments. The intratumoral radiomics model demonstrated higher discriminative ability in the training cohort (AUC = 0.928) compared with the PPAT model (AUC = 0.859), consistent with the expectation that direct tumor imaging contains the most disease-specific information. However, in the validation cohort, the PPAT model exhibited remarkably stable performance (AUC = 0.842, ΔAUC from training = 0.017), while the intratumoral model showed greater performance degradation (AUC = 0.835,ΔAUC from training = 0.093). Importantly, the combined radiomics model (AUC = 0.955 training, 0.850 validation) outperformed either single-tissue model, confirming that tumor and PPAT radiomics provide complementary rather than redundant biological information.

Several factors may explain the different performance stability between these two tissue compartments. Intratumoral radiomics features may be influenced by the spatial mismatch between imaging findings and pathological sampling. In our study, systematic 12-core biopsy without MRI-ultrasound fusion was used, which may result in discordance between biopsy-proven lesions and the dominant MRI-defined tumor region. Such sampling limitations may introduce uncertainty in the biological interpretation of intratumoral radiomic features. In contrast, PPAT segmentation is based on anatomical boundaries and is independent of biopsy location, which may reduce the influence of biopsy-related spatial uncertainty and contribute to greater feature robustness.

Furthermore, prostate tumors exhibit substantial biological heterogeneity at cellular, molecular, microenvironmental, and morphological levels, which may increase the sensitivity of intratumoral radiomic features to variations in segmentation, image acquisition, and cohort composition. Conversely, PPAT represents a relatively consistent anatomical compartment that may provide more stable imaging characteristics across different cohorts and contribute to improved feature reproducibility. Therefore, intratumoral radiomics may capture more disease-specific information, whereas PPAT radiomics may provide microenvironment-related information with greater robustness and generalizability. The integration of both tissue compartments may achieve a balance between predictive discrimination and generalizability, supporting the potential clinical utility of combined radiomics models for BM risk stratification.

### Role of clinical predictors

Our analysis of clinical predictors revealed that most conventional variables had limited independent utility for predicting BM in the multivariate context. Only clinical T stage and Ki-67 expression emerged as statistically significant independent predictors. Ki-67 is a well-established proliferation marker expressed throughout the cell cycle except in the G0 phase. High Ki-67 expression (> 10%) is associated with poor tumor differentiation and adverse clinical outcomes, including shorter disease-free survival, higher rates of biochemical recurrence, and increased risk of distant metastasis (36, 37). Our finding that Ki-67 expression independently predicts BM provides further clinical validation of its prognostic utility. In addition to its role as an independent clinical predictor, Ki-67 expression showed significant associations with multiple MRI radiomic features in our additional analysis. These findings suggest that radiomic features may reflect non-invasive imaging manifestations of tumor proliferative activity and biological heterogeneity. However, radiomic features should be considered complementary imaging biomarkers rather than direct substitutes for Ki-67 assessment, as radiomics captures complex imaging phenotypes that may integrate multiple underlying biological processes. Clinical T stage reflects the local extent of tumor invasion and has been previously identified as an independent predictor of BM (38), consistent with our results.

Notably, although previous studies have reported age, PSA, alkaline phosphatase, and Gleason score as potential predictors of BM (39, 40),these variables did not retain independent significance in our multivariate analysis. In particular, the lack of independent predictive value of Gleason score may appear counterintuitive given its established prognostic role in PCa. Several factors may explain this finding. First, Gleason score was dichotomized at ≥7 versus <7 in our analysis, which may not fully capture the heterogeneous metastatic risk within different ISUP grade groups. Second, Gleason score was correlated with other clinicopathological variables, such as cT stage and Ki-67 expression, which remained significant in the multivariate model; thus, part of the prognostic information provided by Gleason score may have been captured by these correlated factors. Third, biopsy-derived Gleason grading may be affected by sampling limitations, particularly in patients with heterogeneous or multifocal tumors, potentially weakening its association with metastatic status. Finally, the relatively modest sample size (92 BM events) may have limited the statistical power to detect an independent association after adjustment for multiple variables. These findings do not indicate that conventional clinical parameters lack prognostic value; rather, they suggest that imaging-derived biomarkers may provide complementary information beyond established risk factors. By capturing quantitative features associated with tumor heterogeneity and the surrounding microenvironment, handcrafted radiomics extracted from routinely acquired mpMRI may offer additional predictive information for individualized BM risk stratification.

Importantly, the addition of clinical variables (cT stage and Ki-67) to the Rad-score did not significantly improve discriminative performance (DeLong test: p = 0.350 in training and p = 0.288 in validation). This finding indicates that the radiomics features already encode the vast majority of the predictive information. Nevertheless, we elected to retain the clinical variables in the final nomogram for two reasons: (1) they provide clinically interpretable anchors that enhance model transparency and physician trust, and (2) they may contribute to improved calibration and generalizability in external populations where the radiomics features alone may behave differently due to scanner or protocol variations.

### Clinical implications

The proposed model has several potential clinical applications. First, it could serve as a non-invasive complementary risk stratification tool to identify patients with potentially aggressive disease characteristics who may benefit from further diagnostic evaluation, intensified staging strategies, or closer clinical management. The nomogram’s high sensitivity (0.954 in validation) may be advantageous for identifying patients who may warrant additional evaluation, particularly when minimizing missed metastatic disease is clinically important. Second, by enabling more precise risk stratification, the model has the potential to optimize therapeutic strategies and reduce both overtreatment and undertreatment. With the increasing adoption of PSMA PET/CT, which provides improved detection of bone and lymph node metastases compared with conventional imaging approaches, radiomics-based models are unlikely to replace advanced molecular imaging modalities. Instead, the potential value of this model lies in providing additional biological information from routinely acquired mpMRI to complement existing clinical and imaging assessments. Third, the nomogram format provides an accessible, quantifiable, and individualized clinical tool that can be readily integrated into routine clinical workflows.

The notably lower specificity in the validation cohort (0.460) compared with the training cohort (0.831) warrants particular attention. This decline may be partly related to the relatively limited sample size and low events-per-variable (EPV) ratio. Specifically, the validation cohort contained only 22 BM events, resulting in a limited number of outcome events relative to the complexity of the prediction model incorporating multiple radiomics features and clinical predictors (EPV ≈1.7 based on the validation cohort). This limited event number may reduce the stability of performance estimates and increase their sensitivity to variations in case composition. Consequently, the risk estimates derived from the training cohort may not fully generalize to the validation cohort, potentially leading to an increased number of false-positive classifications and the observed reduction in specificity. Although the model retained high sensitivity (0.954) in the validation cohort, indicating its potential ability to identify patients with BM or potentially aggressive disease characteristics, the moderate specificity limits its interpretation as a stand-alone decision-making tool. Instead, the model may be more appropriately considered as a complementary risk stratification tool for identifying patients with potentially aggressive disease who may warrant further metastatic evaluation or closer clinical attention. Therefore, the model should not be interpreted as a replacement for established metastatic evaluation strategies. In clinical practice, the optimal threshold may need to be adjusted according to the specific clinical context: a lower threshold to maximize sensitivity when the cost of missed BM is prioritized, or a higher threshold to improve specificity in settings where the cost of false-positive classifications is prioritized. The threshold selection should ultimately be guided by clinical priorities and cost-effectiveness analyses. In addition to the low EPV ratio, the multi-step feature selection pipeline used in this study—including univariate screening, collinearity reduction, and LASSO regression—was performed within the entire training cohort rather than being embedded within nested cross-validation folds. This approach may introduce a potential risk of information leakage, as outcome information from all training samples contributed to feature selection decisions. Such methodological limitations may have resulted in optimistic performance estimates in the training cohort and, together with the limited number of outcome events, may have contributed to reduced generalizability in the validation cohort. Future radiomics studies should consider nested cross-validation or fully separated feature-selection procedures to obtain more robust estimates of model performance.

### Limitations

Several limitations of this study should be acknowledged. First, the retrospective, single-center design with a relatively small sample size (n = 237; 92 BM events) may introduce selection bias and limit the generalizability of our findings. The relatively high BM prevalence (38.8%) in our cohort, compared with the lower prevalence expected in unselected PCa populations at diagnosis, reflects referral bias at a tertiary center and may lead to overestimation of positive predictive value. Future multicenter, prospective studies with larger and more representative cohorts are essential to validate and refine our model. Second, the events-per-variable (EPV) ratio in the validation cohort was relatively low, with only 22 BM events available for evaluation of a model incorporating multiple radiomics features and clinical predictors (EPV ≈ 1.7). This limited number of positive events may reduce the precision and stability of performance estimates and increase the sensitivity of specificity estimates to case-mix variations. The observed decrease in specificity from 0.831 in the training cohort to 0.460 in the validation cohort may therefore partly reflect reduced stability of model performance estimates associated with the limited number of validation events and potential model overfitting. Although LASSO regularization was applied to reduce model complexity, larger independent cohorts are required to further validate the robustness and generalizability of the proposed model. Third, although intra-observer reproducibility was assessed (ICC ≥ 0.75), inter-observer agreement was not formally quantified. Only one reader performed the segmentation, with a senior radiologist providing confirmatory review. Independent segmentation by multiple readers with formal inter-observer ICC assessment would have strengthened the reproducibility evidence. Fourth, our multi-step feature selection pipeline — including univariate screening, collinearity reduction, LASSO regression with 10-fold cross-validation, and stepwise regression — was applied to the entire training cohort before final model fitting. While this approach is common in the radiomics literature, it may introduce selection bias and increase the risk of optimistic performance estimates. Specifically, the outcome labels (BM status) of all training samples were used indirectly during the feature selection process because the entire training cohort was involved in identifying predictive features. Ideally, feature selection should be embedded within nested cross-validation folds, where the entire selection process is repeated independently in each training fold, and the held-out fold is used only for final model evaluation. By using the full training set for feature selection, our model may have captured cohort-specific correlations that may not fully generalize to independent populations, potentially resulting in optimistic estimates of model performance. This potential bias, together with the limited EPV ratio discussed above, may have contributed to the observed performance degradation between the training and validation cohorts. Future studies should adopt nested cross-validation or a fully separated feature-selection split to obtain more robust and unbiased estimates of model generalizability. Fifth, this study was conducted at a single institution and lacked an independent external validation cohort. Although internal validation was performed, differences in patient characteristics, MRI acquisition protocols, scanner manufacturers, and post-processing procedures across institutions may influence radiomics feature stability and model performance. Therefore, multicenter external validation using heterogeneous imaging datasets is essential to confirm the robustness, reproducibility, and generalizability of the proposed model before clinical implementation. Sixth, the feature extraction software (FAE v0.5.8) has not been formally benchmarked against the Image Biomarker Standardization Initiative (IBSI) standards, which may limit cross-study comparability. Seventh, while most patients underwent mpMRI before biopsy, a small subset of patients underwent MRI after biopsy. Biopsy-related changes, including hemorrhage, inflammation, or edema, may introduce artifacts that could affect radiomic feature extraction, particularly for intratumoral features. Future studies should ideally acquire mpMRI before biopsy to minimize such potential confounding effects. Eighth, we acknowledge an important limitation regarding biopsy–MRI spatial correlation. All biopsies in this study were performed using systematic 12-core transrectal/transperineal biopsy, which was the standard diagnostic approach at our institution during the study period. MRI-ultrasound fusion targeted biopsy was not available at our center at the time of this study. Consequently, we could not systematically confirm whether the positive biopsy cores corresponded spatially to the MRI-defined index lesion from which intratumoral radiomic features were extracted. This spatial uncertainty between imaging findings and pathological sampling, particularly in patients with multifocal disease, may affect the biological interpretation and generalizability of intratumoral radiomic features. Future prospective studies incorporating MRI-ultrasound fusion biopsy are warranted to establish more precise imaging–pathology correlations and further validate radiomics-based biomarkers. Ninth, detailed pathological variables were not comprehensively available in this retrospective cohort. Although Ki-67 expression was incorporated as a pathological biomarker because it was consistently recorded during the study period, other important pathological features, including cribriform morphology and intraductal carcinoma, were not systematically documented and therefore could not be incorporated into the prediction model. Given their established prognostic relevance in prostate cancer, future prospective studies with comprehensive pathological annotation are warranted to determine whether integration of these variables can further improve metastatic risk prediction. Finally, our analysis only included patients with definitive BM status; those with equivocal findings on ECT/PET-CT were excluded. This may limit the model’s applicability to the very population where a non-invasive prediction tool would be most clinically valuable—patients with uncertain bone status.

## Conclusion

In conclusion, we developed and validated a novel combined prediction model integrating mpMRI-based radiomics features from both the prostate tumor and periprostatic adipose tissue with independent clinical predictors (T stage and Ki-67 expression) for predicting bone metastasis in patients with newly diagnosed prostate cancer. The combined model demonstrated encouraging discriminative performance and promising clinical net benefit, offering a promising non-invasive tool for individualized BM risk stratification beyond conventional clinical assessment, although further validation in larger prospective cohorts is required. The integration of PPAT radiomics features alongside intratumoral features represents a biologically motivated and technically feasible strategy that captures complementary information from the tumor and its microenvironment, potentially facilitating the identification of patients with more aggressive disease characteristics who may benefit from further diagnostic evaluation or intensified staging strategies. Future multicenter prospective studies with external validation are warranted to confirm the robustness and clinical utility of this model before its implementation in clinical practice.

## Data Availability

The raw data supporting the conclusions of this article will be made available by the authors, without undue reservation.
