# Supplementary material for: Multiparametric MRI-based radiomics model integrating tumor lesion and periprostatic adipose tissue for predicting bone metastasis in prostate cancer
Source: Front Oncol. 2026 Sep 7;16:1918323. doi: 10.3389/fonc.2026.1918323 (PMC13590420; doi:10.3389/fonc.2026.1918323)
Supplement: Supplementary file 1 [file Table1.docx]

**Supplementary Table S1. Methodological assessment of the present study according to relevant domains of the Radiomics Quality Score 2.0 (RQS 2.0)**

| **RQS 2.0-related domain** | **Implementation in the present study** | **Compliance** |
| --- | --- | --- |
| Clinical objective and study design | Retrospective study designed to develop and internally validate an mpMRI-based handcrafted radiomics model integrating tumor lesion and PPAT features with clinical predictors for prediction of synchronous bone metastasis in newly diagnosed prostate cancer. | Yes |
| MRI acquisition protocol | Multiparametric MRI including T2-weighted imaging, T2WI with fat suppression, and ADC sequences was performed. Imaging parameters were predefined and described in the Methods section. | Yes |
| Image preprocessing | MRI images underwent preprocessing including resampling, intensity normalization, and gray-level discretization before radiomics feature extraction. | Yes |
| Segmentation strategy | Primary prostate lesions and periprostatic adipose tissue (PPAT) regions were manually segmented according to predefined anatomical criteria. | Yes |
| Feature reproducibility assessment | Feature robustness was assessed using intraclass correlation coefficients (ICC), and features with poor reproducibility were excluded before model construction. | Yes |
| Radiomics feature extraction | Handcrafted radiomics features were extracted from tumor lesions and PPAT regions based on multiparametric MRI following IBSI-compliant feature categories. | Yes |
| Feature selection and model development | Feature reduction was performed using univariate analysis, correlation analysis, and LASSO regression. A combined clinical-radiomics model was subsequently constructed. | Yes |
| Integration of clinical predictors | Clinical T stage and Ki-67 expression were incorporated with radiomics features to develop the final nomogram. | Yes |
| Internal validation | The cohort was randomly stratified into training and validation sets using a 7:3 ratio. Internal validation was performed using the independent validation cohort. | Yes |
| Model performance evaluation | Model discrimination, calibration, and clinical utility were evaluated using ROC curves, AUC analysis, Hosmer-Lemeshow calibration testing, calibration curves, and decision curve analysis. | Yes |
| Cutoff determination | The optimal cutoff value was determined using the Youden index to balance sensitivity and specificity. | Yes |
| External validation and generalizability | External multicenter validation was not performed. The limitation of single-center design and lack of external validation was acknowledged. | Not performed |
| Clinical translation | The model is currently considered a complementary risk stratification tool rather than a replacement for established metastatic imaging approaches. Further prospective validation is required before clinical implementation. | Future work |

**RQS 2.0, Radiomics Quality Score 2.0; mpMRI, multiparametric magnetic resonance imaging; PPAT, periprostatic adipose tissue; ADC, apparent diffusion coefficient; T2WI, T2-weighted imaging; ICC, intraclass correlation coefficient; LASSO, least absolute shrinkage and selection operator; BM, bone metastasis; PCa, prostate cancer.**

**TableS2 Radiomic features selection result**

| **ROIs** | **MRI sequences** | **Filter** | **Feature class** | **Feature** |
| --- | --- | --- | --- | --- |
| PPAT | T2WI | Square Root | First-order | Root Mean Squared |
| PPAT | T2WI-FS | Wavelet HLL | First-order | Skewness |
| PPAT | T2WI-FS | LBP 3D m1 | First-order | Skewness |
| PPAT | T2WI-FS | Wavelet HLH | GLDM | Small Dependence Low Gray Level Emphasis |
| Intratumoral region | T2WI | Gradient | First-order | Robust Mean Absolute Deviation |
| Intratumoral region | T2WI | Original | GLSZM | Large Area High Gray Level Emphasis |
| Intratumoral region | T2WI | Wavelet LLL | First-order | Minimum |
| Intratumoral region | T2WI-FS | Wavelet LLL | GLSZM | Large Area High Gray Level Emphasis |
| Intratumoral region | T2WI-FS | Wavelet LLL | NGTDM | Complexity |
| Intratumoral region | T2WI-FS | Wavelet HHH | GLDM | Large Dependence High Gray Level Emphasis |
| Intratumoral region | T2WI-FS | Original | GLDM | Large Dependence Low Gray Level Emphasis |
| Intratumoral region | ADC | Wavelet LLL | First-order | 10th Percentile |
| Intratumoral region | ADC | Original | GLDM | Dependence Variance |

**ROIs=Regions of interests; MRI=Magnetic resonance imaging; T2WI=T2-weighted imaging;T2WI FS=Fat-suppressed T2-weighted imaging;ADC=Apparent diffusion coefficient; GLDM=Gray-level dependence matrix; GLSZM=Gray-level size-zone matrix; NGTDM=Neighboring gray-tone difference matrix; PPAT=periprostatic adipose tissue.**

**Supplementary Table S3. Association between MRI radiomic features and Ki-67 expression status**

| **ROI** | **MRI sequences** | **Feature** | **Negative median** | **Positive median** | **P value** | **FDR** |
| --- | --- | --- | --- | --- | --- | --- |
| Intratumoral region | T2WI | Gradient First-order Robust Mean Absolute Deviation | -0.409 | 0.327 | <0.001 | <0.001 |
| Intratumoral region | T2WI | Original GLSZM Large Area High Gray Level Emphasis | -0.381 | -0.214 | <0.001 | <0.001 |
| Intratumoral region | T2WI | Wavelet-LLL First-order Minimum | 0.241 | -0.144 | <0.001 | <0.001 |
| Intratumoral region | T2WI-FS | Wavelet-LLL GLSZM- Large Area High Gray Level Emphasis | -0.638 | 0.137 | <0.001 | <0.001 |
| Intratumoral region | T2WI-FS | Wavelet-LLL NGTDM Complexity | -0.578 | -0.094 | <0.001 | <0.001 |
| Intratumoral region | T2WI-FS | Wavelet-HHH GLDM Large Dependence High Gray Level Emphasis | -0.457 | -0.129 | <0.001 | <0.001 |
| Intratumoral region | T2WI-FS | Original GLDM Large Dependence Low Gray Level Emphasis | -0.085 | -0.457 | <0.001 | <0.001 |
| Intratumoral region | ADC | Wavelet-LLL First-order 10th Percentile | 0.319 | -0.429 | <0.001 | <0.001 |
| Intratumoral region | ADC | Original GLDM Dependence Variance | -0.552 | 0.180 | <0.001 | <0.001 |
| PPAT | T2WI | Square Root First-order Root Mean Squared | -0.268 | 0.120 | 0.009 | 0.009 |
| PPAT | T2WI-FS | Wavelet-HLL First-order Skewness | 0.294 | -0.151 | 0.002 | 0.002 |
| PPAT | T2WI-FS | LBP 3D m1 First-order Skewness | 0.267 | -0.353 | <0.001 | <0.001 |
| PPAT | T2WI-FS | Wavelet-HLH GLDM Small Dependence Low Gray Level Emphasis | 0.032 | -0.243 | 0.212 | 0.212 |

**ROIs=Regions of interests; MRI=Magnetic resonance imaging; T2WI=T2-weighted imaging;T2WI FS=Fat-suppressed T2-weighted imaging;ADC=Apparent diffusion coefficient; GLDM=Gray-level dependence matrix; GLSZM=Gray-level size-zone matrix; NGTDM=Neighboring gray-tone difference matrix; PPAT=periprostatic adipose tissue; P values were calculated using the Mann–Whitney U test; FDR=false discovery rate adjusted using the Benjamini–Hochberg method**
